# Unsupervised Representation Learning for Proteochemometric Modeling

**DOI:** 10.3390/ijms222312882

**Published:** 2021-11-28

**Authors:** Paul T. Kim, Robin Winter, Djork-Arné Clevert

**Affiliations:** Bayer Machine Learning Research, Müllerstraße 178, 13353 Berlin, Germany; robin.winter@bayer.com

**Keywords:** unsupervised representation learning, computational biology, protein–ligand binding prediction

## Abstract

In silico protein–ligand binding prediction is an ongoing area of research in computational chemistry and machine learning based drug discovery, as an accurate predictive model could greatly reduce the time and resources necessary for the detection and prioritization of possible drug candidates. Proteochemometric modeling (PCM) attempts to create an accurate model of the protein–ligand interaction space by combining explicit protein and ligand descriptors. This requires the creation of information-rich, uniform and computer interpretable representations of proteins and ligands. Previous studies in PCM modeling rely on pre-defined, handcrafted feature extraction methods, and many methods use protein descriptors that require alignment or are otherwise specific to a particular group of related proteins. However, recent advances in representation learning have shown that unsupervised machine learning can be used to generate embeddings that outperform complex, human-engineered representations. Several different embedding methods for proteins and molecules have been developed based on various language-modeling methods. Here, we demonstrate the utility of these unsupervised representations and compare three protein embeddings and two compound embeddings in a fair manner. We evaluate performance on various splits of a benchmark dataset, as well as on an internal dataset of protein–ligand binding activities and find that unsupervised-learned representations significantly outperform handcrafted representations.

## 1. Introduction

A main goal of cheminformatics in the area of drug discovery is to model the interaction of small molecules with proteins in silico. The ability to accurately predict the binding affinity of a ligand towards a biological target without the need to conduct costly and time-consuming in vitro experiments has the potential to accelerate drug development processes by enabling early prioritization of promising drug candidates [1]. A common approach is to train a machine learning algorithm to predict the binding affinity of ligands towards a certain biological target by using a training set of compounds that have been experimentally measured on this target. This modality is commonly referred to as a quantitative structure–activity–relationship (QSAR) model and uses the similarities and differences between molecules, represented in various ways, in order to learn patterns about their properties [2].

**Figure 1:**
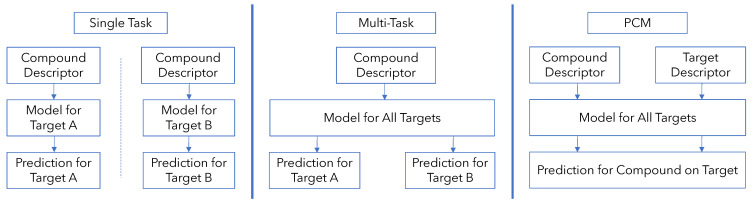
Comparison of single-task, multi-task and proteochemometric modeling strategies for protein–ligand binding prediction.

QSAR models can be broadly classified into two types: single-task QSAR models and multi-task QSAR models (Figure 1). In single-task QSAR modeling, a model is trained separately for each protein to predict a binary or continuous outcome (binding vs. not-binding or the binding affinity) given a compound input. The machine learning model used could be anything from simple linear models to tree-based and random forest methods to deep neural networks [3,4]. In multi-task modeling, a single model is trained to predict binding across multiple proteins simultaneously, allowing the model to take advantage of the correlations in binding activity between compounds on different targets [5,6]. This is performed, for example, by using a neural network with multiple output nodes where each output node corresponds to a different protein. Thus, multiple outputs are predicted given a compound input [7,8].

While these methods have been employed on various protein targets and are particularly useful when total data are limited to only a few targets, there are two major methodological limitations to their use [9,10]. Both single-task and multi-task models must be retrained from scratch if one wishes to incorporate binding data for a new protein, and they cannot be used to make predictions on new protein targets for which experimental data are absent [2].

An attractive solution to this problem is to also include protein information to the input in a so-called proteochemometric (PCM) model (Figure 1). The additional protein information enables a PCM model to directly utilize similarities between proteins for bioactivity modeling [1,2]. With an expressive protein descriptor, a model could leverage similarities and differences between proteins directly to model their binding behaviors rather than merely using correlations found among the compounds they bind to, as in multi-task modeling, or ignoring protein relationships altogether as in single-task modeling [1,2]. Consequently, PCM models have found success in a variety of protein targets and using many different machine learning methods, including random forests (RF) and support vector machines (SVM) [11,12,13,14,15].

Recent advances in the field of deep learning have also resulted in its use in QSAR single and multi-task modeling as well as PCM modeling [3,16,17,18]. Lenselink et al. [3] compared deep learning methods against other machine learning methods for PCM, single-task QSAR and multi-task QSAR models on a benchmark dataset, and the authors found that PCM models using deep neural networks outperformed other machine learning methods as well as single-task and multi-task QSAR models.

All of the aforementioned studies utilize features for both small molecules and proteins based on hand-crafted feature extraction protocols. For small molecules, the most widely used method of representation is chemical fingerprints based on substructure presence or substructure counts [2,19,20]. As the number of potential substructures is vast, usually generated procedurally to approximately ∼2^32^ substructures, the resulting sparse set of bits is usually hashed and folded to a much smaller size (∼10^3^) at the expense of hash and bit collisions [20]. These structure-based descriptors can also be augmented with physicochemical descriptors [21,22].

Meanwhile, the most commonly used handcrafted protein descriptors are computed by amino-acid based or sequence-based methods. For amino-acid based descriptors, physicochemical structure [13,23,24] and (or) 3D-properties [25,26]—or the first principle components of a set of these properties—are aggregated over the entire sequence. Sequence-based descriptors usually involve one-hot encoding of mutated sequence positions found by aligning sequences or by encoding the presence of motifs and sequence patterns [27,28].

In many application domains of deep learning, recent research has shown that these methods generally work better when the input data representation is of a lower level and unabstracted, allowing the model to learn hierarchical features directly rather than relying on features that are hand-crafted by humans [17,18]. For example, this is the case in computer vision, where deep learning on pixel value features has been the state of the art for several years [18]. In cases where the input space is too high-dimensional and especially if there are not enough labeled data to train a model end-to-end, unsupervised representation learning is used to generate lower-dimensional representations, known as “embeddings,” because they “embed” a data point into a lower-dimensional space. These embedding methods also rely on machine learning, rather than human engineering, for feature extraction. These techniques are used in a variety of machine learning tasks, including natural language processing, as well as video analysis [29,30,31].

In this study, we follow this reasoning and utilize unsupervised and self-supervised-learned embeddings to represent both ligand and protein spaces for proteochemometric modeling. We hypothesize that proteochemometric modeling might benefit from such embeddings, since the number of labeled data points, i.e., measured ligand-target binding affinities, are scarce, but the number of unlabeled data points, i.e., molecular structures and protein sequences, are vast.

We compare the performances of two compound embeddings and three protein embeddings, described further in the following section, on a benchmark dataset. We also compare performance to a No-Interaction-Terms model, which does not allow information flow between protein and compound input representation, in order to analyze the properties of these embeddings in a PCM model. Finally, we train and test models using these unsupervised descriptors on a large internal protein–ligand activity dataset.

## 2. Methods

In the following section, we will discuss the compound and protein embeddings tested in our experiments, describe the datasets and types of train-valid-test splits utilized, provide details on the deep learning models trained and explain the metrics used to evaluate performance.

### 2.1. Embeddings

For compound embeddings, we use the Continuous and Data Driven Descriptors (CDDD), developed by Winter et al. [32], and MolBert (Molecule-BERT), developed by Fabian et al. [33]. CDDD and MolBert descriptors offer unique, compact and continuous vector representations for each compound, as opposed to fingerprints, which are non-unique, discrete and must be hashed to be made compact. Molecules with the same substructures but that are differently arranged will correspond to different vectors. CDDD uses a recurrent autoencoder trained on the reconstruction task, while MolBERT uses a Transformer architecture trained on three self-supervised tasks. These unsupervised-learned descriptors have demonstrated competitive or superior results compared to molecular fingerprints on a variety of other tasks, indicating their ability to effectively represent compound properties and behaviors [32,33].

For protein embeddings, we use embeddings generated by UniRep [34], SeqVec [35] and the Evolutionary Scale Model (ESM) [36], which are language models trained on protein sequences in an unsupervised manner. Similarly to compound embeddings, these protein embeddings offer unique, compact and continuous vector representations of proteins. These methods all train language models on UniRef 50, a very large and non-redundant corpus of protein sequences, but differ in key implementation details. UniRep learns 256-length embeddings by averaging over the hidden states of a multiplicative LSTM model (a variant of the LSTM, abbreviated mLSTM) [37] that are produced as the model predicts the next amino acid of a sequence [34]. SeqVec takes a similar approach but uses a larger and deeper model—an adapted version of the ELMo language model—which contains stacked convolutional and recurrent layers [35]. The 1024-length embedding is generated after summing across the layers and averaging across sequences. The ESM, on the other hand, uses a very large Transformer model [38] and trains on the masked language model task, thus using self-attention to learn dependencies between masked sequence positions and the rest of the (unmasked) sequence. ESM is by far the largest model of the three, containing 650 M parameters and 33 attention layers [36]. A summary of both the compound and protein descriptors, including the number of parameters used and the size of the vector representations, can be found in Table 1.

All the descriptors discussed have been shown to be useful for many different protein-related prediction tasks, including secondary structure and contact prediction, as well as remote homology detection. However, to the best of our knowledge, this is the first study that utilizes these unsupervised-learned protein and compound embeddings for the purpose of training a protein–ligand binding prediction model.

### 2.2. Dataset and Evaluation

We evaluate the performance of different descriptors on a large-scale benchmark PCM dataset created in Lenselink et al. [3], which contains 310 k compound–protein bioactivity measurements exclusively taken from the highest-confidence bioactivity assay data in ChEMBL, a database of drug-like bioactive molecules maintained by the European Molecular Biology Laboratory (EMBL). The dataset comprises 1226 unique human proteins from a range of protein families and 190 k unique compounds. In the following, we shall refer to this dataset as “ChEMBL.” Additionally, we report results on a large internal dataset of protein–ligand binding activities, containing 500 k unique compounds and 1 k unique proteins. This dataset shall be referred to as “Internal” in the following sections.

In order to evaluate performance of the representations, we use three different types of hold-out-sets. On the ChEMBL dataset, we use random leave-compound-cluster-out and leave-protein-out splits, while on Internal, we used only leave-compound-cluster-out splits. Below, we will describe the difference between these splits generally. Details on how these splits were implemented can be found in Appendix A.

Random splits randomly divide bioactivity measurements into train, valid and test sets. A drawback of the random split is that it can assign bioactivity measurements from the same experimental assay into the training and test sets. Since a single assay can involve similar, congeneric compounds, it is likely that there will be compounds in the training set that are highly similar to compounds in the test set and measured on the same protein. This split can, thus, report over-optimistic results [3].

Leave-compound-cluster-out (LCCO) splits directly address the issue of compound similarity between training and test sets. For these splits, compounds are divided into several clusters based on a pairwise distance metric. Then, one cluster is held out as a test set, while the remaining clusters are used for training and validation; this process can be repeated for each cluster to perform multi-fold cross-validation over the entire dataset. Since the test set explicitly contains chemical matter that is, according to the distance metric, distinct from the training set, a model’s performance on this split is a better proxy for its ability to generalize to unseen regions of the compound space compared to random splits.

Leave-protein-out (LPO) splits instead test the ability of the model to generalize to new proteins. This is very challenging, as while the model can show hundreds of thousands of unique compounds, it can only be shown thousands of unique proteins with currently available datasets. Nevertheless, this is a problem mode that single-task and multi-task models cannot operate on by construction; therefore, it is interesting to test if the PCM models can learn to generalize to new proteins in this manner.

### 2.3. Development of Models

In order to make a fair comparison between the different descriptor combinations, we aimed to use a “neutral” model and hyperparameter settings. Based on previous work [3,41], we knew that a simple feedforward neural network would be well suited for this task. For model architecture, we used a modified version of our DeepPCM network [41], which is a feedforward neural network containing 6 M parameters in total across 3 layers. For implementation details, refer to Appendix B.

For all of our experiments, we also used a standard hyperparameter setup that is similar to the setup used in [3]. We use an Adam optimizer with default learning rate and parameters [42], learning rate halving upon validation performance plateau and early stopping after ten epochs without improvement.

We did not tune the model architecture or hyperparameters on any dataset because we are more interested in the relative performance of these descriptors rather than absolute maximal performance.

In addition, we use a No-Interaction-Terms model as a baseline to compare against the “full” PCM model. The No-Interaction-Terms model keeps compound and protein channels separate throughout the network and makes the final model prediction by taking the average of the separate predictions made from compound and protein information—otherwise, it has an identical architecture as the “full” PCM model. We can investigate the difference between this model, which can at best only recover compound and protein bias in the dataset, and the full model, which is potentially able to model the specific bio-chemical interaction between the protein and the ligand. A diagram is available in Appendix B.

### 2.4. Model Quality Evaluation Metrics

The descriptors are compared by using the Matthews Correlation Coefficient (MCC) and Boltzmann-Enhanced ROC (BEDROC) [3]. The MCC score represents overall model quality and is especially useful for measuring performance on unbalanced datasets. The BEDROC score is a metric that represents the effectiveness of the model for compound prioritization; since only a small subset of in silico screened compounds often can be tested experimentally, a useful model will rank active compounds very highly [43]. The BEDROC score represents this by weighting the ROC results such that 80% of the BEDROC score comes from the top 8% of predicted actives. Thus, it is analogous to an ROC-50 score.

## 3. Results and Discussion

On random and leave-compound-cluster-out splits on ChEMBL, the models that use unsupervised-learned descriptors perform well, particularly attaining MCCs of around 0.5 and BedROCs of around 0.94 on the leave-compound-cluster-out split. In concrete terms, by examining the MolBert and UniRep-based model, for example, on the leave-compound-cluster-out split, we observed on average a 25% overlap between the top 10% highest activity compounds and top 10% highest predicted activity compounds for each protein (ignoring proteins with fewer than 100 total tested compounds, for which such metrics would not be meaningful).

Our results are consistent with our hypothesis that the descriptors generated via unsupervised representation learning are more powerful than handcrafted protein and compound descriptors. Across all splits and models, unsupervised descriptors outperform handcrafted ones to a statistically significant degree. Additionally, on the random and leave-compound-cluster-out splits on ChEMBL, we observed that models that use MolBert to represent molecules significantly outperformed models that use the CDDD, highlighting the power of Transformer models for extracting meaningful representations of compounds. Similarly, on those splits, models using UniRep significantly outperformed models using ESM or SeqVec protein descriptors (Table 2). For full results on these splits, see Supplementary Tables S1–S3.

Performance on Internal, on which the leave-compound-cluster-out split was applied, improves relative to the performance on ChEMBL, which is expected as the model now has more data to learn from. Additionally, the finding that MolBert + UniRep is a good descriptor combination to use for the protein–ligand binding prediction task is replicated, as we find that this combination significantly outperforms all other combinations, except for MolBert + ESM (Table 3).

The small but significant improvement of using UniRep relative to SeqVec and ESM is surprising, especially because SeqVec and UniRep are both trained on the same next-character-prediction task and on the same protein corpus (Table 1). We hypothesize that UniRep performs better on this task because it encodes a much more compact features space (256-length vs. 1024 or 768-length) and/or because the mLSTM architecture used by UniRep requires less pooling operations to arrive at the final protein-level representation. SeqVec must pool both over LSTM hidden layers and the sequence dimension of the protein, and ESM must pool over many attention outputs and the sequence dimension, but UniRep only has a mLSTM layer to pool over.

On the leave-protein-out split, although the unsupervised-learned descriptors perform significantly better than the handcrafted descriptors, the overall performance is much worse compared to the random or leave-compound-cluster-out splits. There are many reasons why this may be the case—our dataset may simply contain insufficient proteins for protein-space generalization to be possible, or the protein descriptors might struggle in capturing some of the relevant information for binding, since they are trained on sequences and not on 3D-structure or binding site information. Additionally, the leave-protein-out split is more difficult because it requires the model to generalize both unseen proteins and new compound clusters. Compounds tested on a given protein are often specific to that protein, and so splitting by holding out all compound–protein activities for a given protein inadvertently can also hold out compound clusters. We can detect this by observing the average distance of each test-set compound to the nearest compound in the training set, as measured by cosine distance between MolBert representations. On the random split, the average distance from each test set compound to its nearest compound in the training set is 0.026; on the leave-compound-cluster-out split, it is 0.14; and for the leave-protein-out split, it is 0.085. Thus, the leave-protein-out split is made even more difficult because it is also indirectly testing compound-cluster generalization.

Furthermore, we compare the performance of the model on all splits with the performance of the No-Interaction-Terms model (Table 4). For full results, see Supplementary Tables S4–S6. The No-Interaction-Terms model performs significantly worse than the full model on all splits. This is consistent with our expectation that the full model is able to make predictions based on specific protein–ligand interaction information and is not merely collapsing to predict the protein or compound’s independent propensity for binding on the dataset, which is what the No-Interaction-Terms model is restricted to learning. By comparing performance improvement when using the full model versus the No-Interaction-Terms model between splits, we find that the full model improves performance over the No-Interaction-Terms model by, on average, 10% on the random split ($p=3.1*10^{-10}$), 15% on the compound-cluster-out split ($p=1.7*10^{-11}$) and 7% on the protein-out-split ($p=1.8*10^{-8}$). This suggests that on the compound-cluster-out split, the ability of the model to learn protein–ligand interaction terms assists with generalizing unseen compound clusters. Additionally, this generalization power is not as useful on the random split; thus, the improvement of the full model over the No-Interaction-Terms model is lower on this split. Meanwhile, the comparatively minor improvement of the entire model over the No-Interaction-Terms model on the leave-protein-out split indicates that it is more difficult to learn interaction terms that can generalize well with respect to new proteins.

## 4. Conclusions

In summary, the results show that unsupervised-learned descriptors offer significant improvements over handcrafted descriptors in Proteochemometric modeling (PCM). Additionally, we observed that the Transformer-based MolBert descriptors performed the best with respect to representing compounds, while for protein descriptors, the choice of which of the three unsupervised-learned descriptor generators to use appears to be less significant. By comparing performance of the full model to a No-Interaction-Terms model, we were able to confirm that PCM formulation allows the model to learn relevant interaction terms between ligand and target and is not merely reflecting the bias in the dataset. We believe that comparing to such a baseline should be standard practice when evaluating the performance of PCM models. While the model is effective at generalizing unseen compound clusters, generalizing unseen proteins remains a challenge—this may be improved by generating more data with more unique proteins and by developing more powerful protein descriptors that contain binding site information, which we believe are the most relevant directions for future research in this area.

## Figures and Tables

**Table 1:** Marginal performance (average of all models containing this descriptor on this task) and additional information for different descriptors.

	Marginal Performance			Model Info		
	Random MCC	LCCO MCC	LPO MCC	Repr Size	# Params	Type
CDDD	0.610	0.483	0.309	512	26 M	GRU-Autoencoder
MolBert	**0.630**	**0.497**	0.306	768	85 M	Transformer
UniRep	**0.649**	**0.498**	0.306	256	1.8 M	m-LSTM
SeqVec	0.591	0.481	0.317	1024	93 M	Stacked LSTM
ESM	0.629	0.492	0.296	1280	650 M	Transformer

Abbreviations: See Table 2. Random MCC: MCC on Random Split; LCCO MCC: MCC on LCCO Split; LPO MCC: MCC on LPO Split; Repr Size: Representation Size; Params: Number of model parameters; GRU: Gated-Recurrent-Unit Model [39]; LSTM: Long-and-Short-Term Memory Network [40]. Bold indicates statistically significant performance improvement by wilcoxon signed-rank test over data splits.

**Table 2:** Results on test set for model with different descriptor combinations on benchmark ChEMBL dataset. Best results are denoted in bold. Standard deviations of metrics are shown in parentheses. Raw results for each split can be found in Supplementary Tables S1–S3.

	Random		LCCO		LPO	
	MCC	BedROC	MCC	BedROC	MCC	BedROC
CDDD + UniRep	0.645 (0.004)	0.979 (0.002)	0.490 (0.061)	0.941 (0.017)	0.307 (0.031)	0.847 (0.038)
CDDD + SeqVec	0.575 (0.079)	0.967 (0.012)	0.475 (0.060)	0.930 (0.021)	**0.322**(0.028)	**0.851** (0.041)
CDDD + ESM	0.609 (0.014)	0.974 (0.003)	0.484 (0.054)	0.930 (0.023)	0.297 (0.093)	0.834 (0.093)
MolBert + UniRep	**0.654** (0.005)	**0.980** (0.002)	**0.505** (0.053)	**0.943** (0.018)	0.312 (0.024)	0.847 (0.040)
MolBert + SeqVec	0.607 (0.030)	0.973 (0.007)	0.487 (0.062)	0.938 (0.017)	0.311 (0.035)	0.842 (0.049)
MolBert + ESM	0.630 (0.009)	0.977 (0.002)	0.499 (0.053)	0.937 (0.022)	0.294 (0.118)	0.832 (0.090)
Handcrafted	0.337 (0.003)	0.819 (0.007)	0.276 (0.024)	0.753 (0.058)	0.132 (0.051)	0.655 (0.061)

Abbreviations: CDDD: Continuous and Data Driven Descriptors; UniRep: [34]; SeqVec: [35]; ESM: [36]; MolBert: [33]; MCC: Matthews Correlation Coefficient; BedROC: Boltmann-Enhanced ROC; Random: Random Split; LCCO: Leave-Compound-Cluster-Out; LPO: Leave-Protein-Out. Bold indicates statistically significant performance improvement by wilcoxon signed-rank test over data splits.

**Table 3:** Results on a test set for model with different descriptor combinations on LCCO split of large Internal bioactivity dataset. Best results are denoted in bold. Standard deviation of metrics are shown in parentheses.

	MCC	BedROC
CDDD + UniRep	0.633 (0.021)	0.931 (0.023)
CDDD + SeqVec	0.626 (0.016)	0.922 (0.022)
CDDD + ESM	0.634 (0.023)	0.927 (0.022)
MolBert + UniRep	**0.645** (0.013)	**0.936** (0.018)
MolBert + SeqVec	0.639 (0.012)	0.928 (0.021)
MolBert + ESM	0.646 (0.021)	0.932 (0.021)

Abbreviations: see Table 2.

**Table 4:** Table comparing test set performance of full model to No-Interaction-Terms model various splits of the ChEMBL dataset. Raw results for each split can be found in Supplementary Tables S1–S3.

	Random			LCCO			LPO		
	No-Int	Full	% Imp	No-Int	Full	% Imp	No-Int	Full	% Imp
CDDD + UniRep	0.565	0.645	14.3	0.424	0.490	15.6	0.281	0.307	9.3
CDDD + SeqVec	0.548	0.575	4.9	0.411	0.475	15.6	0.287	0.322	12.2
CDDD + ESM	0.557	0.609	9.4	0.416	0.484	16.3	0.287	0.297	3.5
MolBert + UniRep	0.574	0.654	13.8	0.439	0.505	15.0	0.283	0.312	10.2
MolBert + SeqVec	0.558	0.607	8.7	0.430	0.487	13.3	0.290	0.311	7.2
MolBert + ESM	0.567	0.630	11.2	0.434	0.499	15.0	0.292	0.294	0.7

Abbreviations: see Table 2; No-Int: No-Interaction-Terms Model; Full: Full Model; % Imp: Percentage improvement of Full Model over No-Interaction-Terms Model.

**Figure A1:**
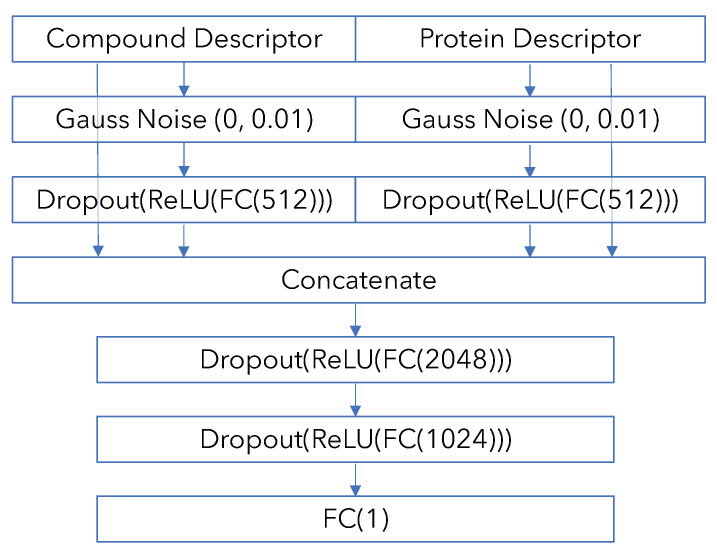
PCM model diagram.

**Figure A2:**
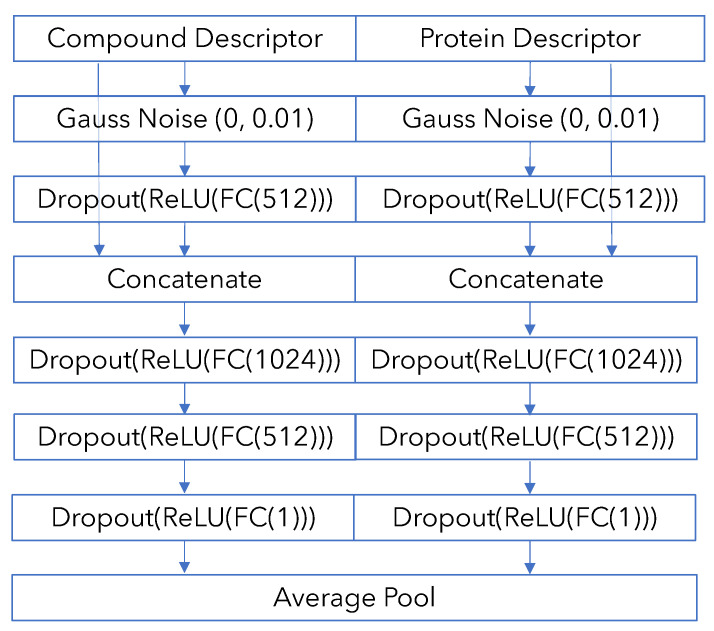
No-Interaction-Terms model diagram.

## Data Availability

The benchmark ChEMBL Dataset of bioactivites used in this study can be found at https://data.4tu.nl/articles/dataset/Beyond_the_Hype_Deep_Neural_Networks_Outperform_Established_Methods_Using_A_ChEMBL_Bioactivity_Benchmark_Set_version_1_/12694478 (accessed on 25 November 2021).

## Supplementary Materials

The following are available online at https://www.mdpi.com/article/10.3390/ijms222312882/s1.

## Appendix A. Data Splitting Implementation Details

For the random splits, we split protein–ligand bioactivities randomly into training, validation and test sets. Ten percent of all bioactivities were added to the test set, and then the remaining 90% of bioactivites were split 70–30 into the training and validation sets, resulting in an overall data split of 63–27–10% train-valid-test. This process was repeated 25 times to create 25 folds, each containing different train, valid and test sets. For leave-compound-cluster-out splits, we took Morgan Fingerprint (256-bit) representations of the compounds and applied k-means (k = 10) to divide the data into ten compound clusters. Then, we created ten folds, and in each fold of the test set, all bioactivities involving compounds in one of the ten compound clusters generated by k-means are included, with the remaining bioactivites being split randomly at 90–10% into training and validation sets. For the leave-protein-out split, we randomly divided all proteins into ten groups. Then, we created ten folds, where in each fold the test set comprised all bioactivites involving proteins in one of the groups, with the training and validation set for each fold being generated from a random 90–10% split of the remaining bioactivites.

## Appendix B. Model Architecture and Training Details

Model adapted from Lenselink [3] and Kim [41]. Training was performed by using the default Adam optimizer [42], a batch size of 512 and a default learning rate scheduler which halves the learning rate upon validation performance plateau. A dropout with *p* = 0.25 was used throughout.
